# Variation in leaf functional traits of *Pinus armandii* and their drivers along an altitudinal gradient in Karst mountains

**DOI:** 10.3389/fpls.2025.1707246

**Published:** 2025-11-06

**Authors:** Bin He, Wangjun Li, Xiaolong Bai, Shun Zou, Qing Li

**Affiliations:** 1 College of Ecological Engineering, Guizhou University of Engineering Science, Bijie, Guizhou, China; 2 Guizhou Province Key Laboratory of Ecological Protection and Restoration of Typical Plateau Wetlands, Bijie, Guizhou, China

**Keywords:** *Pinus armandii*, leaf functional trait, elevational gradient, soil factors, Karst mountains

## Abstract

**Introduction:**

Leaf functional traits are pivotal indicators of plant ecological strategies, reflecting adaptations to environmental conditions. However, the patterns of intraspecific trait variation along environmental gradients and their underlying drivers remain inadequately explored, particularly in fragile ecosystems like karst landscapes.

**Methods:**

We investigated 12 leaf functional traits (encompassing morphological and chemical characteristics) of *Pinus armandii* along an elevational transect (2128 to 2509 m) in the Karst mountainous region of southwestern China. Using correlation and redundancy analyses, we examined altitudinal trends in trait variation and their relationships with key soil factors.

**Results:**

Our results revealed substantial intraspecific variability in all leaf traits, with coefficients of variation ranging from 3.24% to 28.15%. Specific leaf area, leaf length, thickness, area, carbon content, potassium content, and the ratios of C:N, C:P, and N:P decreased significantly with increasing elevation. Conversely, leaf dry matter content, nitrogen content, and phosphorus content increased significantly. We found notable coordination and trade-offs among traits, forming an integrated network centered on the C:N ratio. Soil factors—specifically soil organic carbon, pH, and available potassium—were identified as the primary drivers of this trait variation.

**Discussion:**

*P. armandii* in karst mountainous regions adapts to elevational changes through coordinated adjustments in leaf functional traits, thereby optimizing resource acquisition and use strategies. These findings advance our understanding of plant adaptation mechanisms in such fragile environments.

## 1 Introduction

Global change is rapidly and extensively altering species distributions, biodiversity, and ecosystem functioning (Batllori et al., 2020; Chapin et al., 2000), with profound implications for human well-being (Wang et al., 2021). These changes reshape biodiversity through extinctions, range shifts, and population fluctuations, thereby profoundly altering ecosystem functions (Cardinale et al., 2012; De Laender et al., 2016). Predicting these changes is crucial for conservation and restoration, yet remains challenging due to species diversity and ecosystem complexity. Plant persistence under rapid climate change hinges on adaptive capacity through phenotypic plasticity or genetic change (Nicotra et al., 2015). Consequently, understanding how plant traits respond plastically or genetically along existing environmental gradients is essential for predicting *in situ* persistence under climate change (Chevin and Lande, 2010; Anderson and Gezon, 2015).

Plant functional traits-morphological, physiological, or phenological characteristics-directly reflect plant survival and resource-use strategies across environmental gradients, revealing evolutionary trade-offs and ecological strategies from physiology to ecosystem function (Wright, 2010; Kumari et al., 2021). As primary sensors of global change, plants dynamically adjust traits (e.g., leaf morphology, nutrient allocation) in response to climatic stressors, serving as indicators of ecosystem resilience (Wang et al., 2020; Zhang et al., 2020). Among plant functional traits, leaf functional traits (LFTs), the direct plant-atmosphere interface, are particularly sensitive diagnostic tools. Traits like specific leaf area (SLA), leaf nitrogen content (LNC), leaf dry matter content (LDMC), and photosynthetic capacity are highly sensitive to temperature, moisture, soil nutrients, and altitude (Klich, 2000; Wright et al., 2004; Ordoñez et al., 2009; Ni et al., 2022), elucidating resource acquisition strategies (e.g., leaf economics spectrum) and evolutionary responses to pressures like drought or nutrient limitation (Wright et al., 2004; Reich et al., 2003; Akram et al., 2023). While phylogeny influences LFTs, environmental filters dominate regional trait variation, especially in dynamic habitats (Akram et al., 2023; Liu et al., 2013), underscoring the need to resolve trait responses at the species and population level to scale ecological insights (Navas et al., 2010). Thus, studying PFTs, particularly LFTs, provides a powerful framework for deciphering plant-environment interactions and predicting ecosystem responses to global change (Webb et al., 2010; Díaz et al., 2016; Wang et al., 2023).

Plant functional traits variation underpins species coexistence, community assembly, and ecosystem processes. Intraspecific trait variation (ITV), driven by phenotypic plasticity and local adaptation, constitutes a significant component (up to 30-40%) of total trait variation (Albert et al., 2010; Kattge et al., 2011). ITV critically influences species’ niche breadth, adaptive capacity (Ackerly and Cornwell, 2007), community stability, and ecosystem functioning (Violle et al., 2012; Westerband et al., 2021). However, methodological constraints often lead researchers to rely on species mean traits, potentially obscuring individual-level responses (Westerband et al., 2021). ITV is heterogeneous along environmental gradients like elevation, arising from divergent life forms (Frey et al., 2016; Körner, 2007), ontogenetic plasticity (Siefert et al., 2015), variable precipitation-elevation relationships (Körner, 2007; Martin and Asner, 2009), and evolutionary divergences (Wellstein et al., 2017). Traits also exhibit differential plasticity: structural traits (e.g., SLA, LT) often show high ITV tracking environmental filters, while others (e.g., wood density) display constrained variation due to genetic limitations or counter-gradient responses (Siefert et al., 2015; Umaña et al., 2018). This context-dependency explains inconsistent ITV patterns across studies. A key unresolved question is the link between ITV and individual performance along gradients: whether ITV reflects local adaptation towards an optimum or serves as phenotypic buffering to maintain performance across conditions (Körner, 1991). Therefore, analyzing multi-trait ITV within species across gradients is vital for scaling eco-physiological processes, elucidating assembly mechanisms, and forecasting biodiversity responses.

Mountain ecosystems, characterized by steep environmental gradients, provide unparalleled natural laboratories for studying plant functional responses (Körner, 2007; Graae et al., 2012). Elevational gradients induce systematic shifts in temperature, radiation, moisture, and soil conditions, imposing strong selective pressures that drive intraspecific variation in LFTs linked to resource strategies (Violle et al., 2007; Sides et al., 2014). Typically, lower elevations favor resource-acquisitive traits (e.g., higher SLA), while higher elevations typically select for conservative strategies (e.g., reduced SLA, increased LDMC) (Callaway et al., 2002; Poorter et al., 2009; Pfennigwerth et al., 2017). However, trait responses exhibit significant complexity, showing species specificity, phylogenetic constraints, and functional group divergence (He et al., 2023). Empirical findings for core traits (e.g., SLA, LNC) are often inconsistent across studies, showing increases, decreases, or no trend with elevation (Melis et al., 2023; Xu et al., 2023; Zhao et al., 2016). This uncertainty reflects the context-dependency of trait-environment relationships, modulated by local heterogeneity (topography, soil), biotic interactions (Rasmann et al., 2014), stand factors, and soil properties (e.g., N/P availability, pH) (Liu et al., 2021b; Yang et al., 2023). Furthermore, the mechanisms driving individual LFT responses and trait-trait covariation remain poorly resolved (Onoda et al., 2017). Disentangling these interactions is essential for predicting plant responses to global change and impacts on ecosystem functions (De Deyn et al., 2008; Rixen et al., 2022).

Karst mountains present unique “rock-water-soil-biota” continua with slow soil formation, shallow lithic soils, and high heterogeneity (Wang and Li, 2007). Plants here face dual stressors: seasonal drought and nutrient limitation (Liu et al., 2021a), potentially driving divergent trait strategies compared to other ecosystems. Bedrock exposure may override temperature-driven patterns (He et al., 2020), while calcareous soils (high Ca, low P) exacerbate nutrient constraints (Zhang et al., 2021). Although studies have compared traits between life forms (e.g., evergreen *vs*. deciduous) or karst/non-karst regions (Jiang et al., 2016; Fu and Sun, 2022), and assessed soil influences (Pan et al., 2011), the relative contributions of plant characteristics versus environmental factors to trait variation in these heterogeneous systems remain unclear. Furthermore, existing studies often focus on single-trait variation, neglecting multi-trait coordination and stoichiometric interactions (Han et al., 2023). *Pinus armandii*, a pioneer species in ecological restoration across southwestern China’s karst regions, is vital for ecosystem stability. Despite research on its population dynamics (Yao et al., 2020), ecophysiology (Li et al., 2025), distribution (Liu et al., 2021a), and nutrient limitations (Dong et al., 2022), the elevational patterns of leaf functional trait covariation and their driving mechanisms in karst habitats remain poorly understood. Here, we investigate *P. armandii* along a 2128–2509 m elevational gradient in Guizhou’s karst mountains. By systematically measuring leaf morphological and chemical traits, coupled with soil-climate monitoring, we address: (1) How do LFTs of *P. armandii* vary along elevational gradients in karst habitats? (2) How are morphological traits coordinated with stoichiometric and chemical traits? (3) What are the relative roles of soil properties in driving trait covariation? Our findings will advance understanding of karst plant adaptation, guide restoration species selection, and refine models of leaf trait responses to climate change.

## 2 Materials and methods

### 2.1 Study area

**Figure 1:**
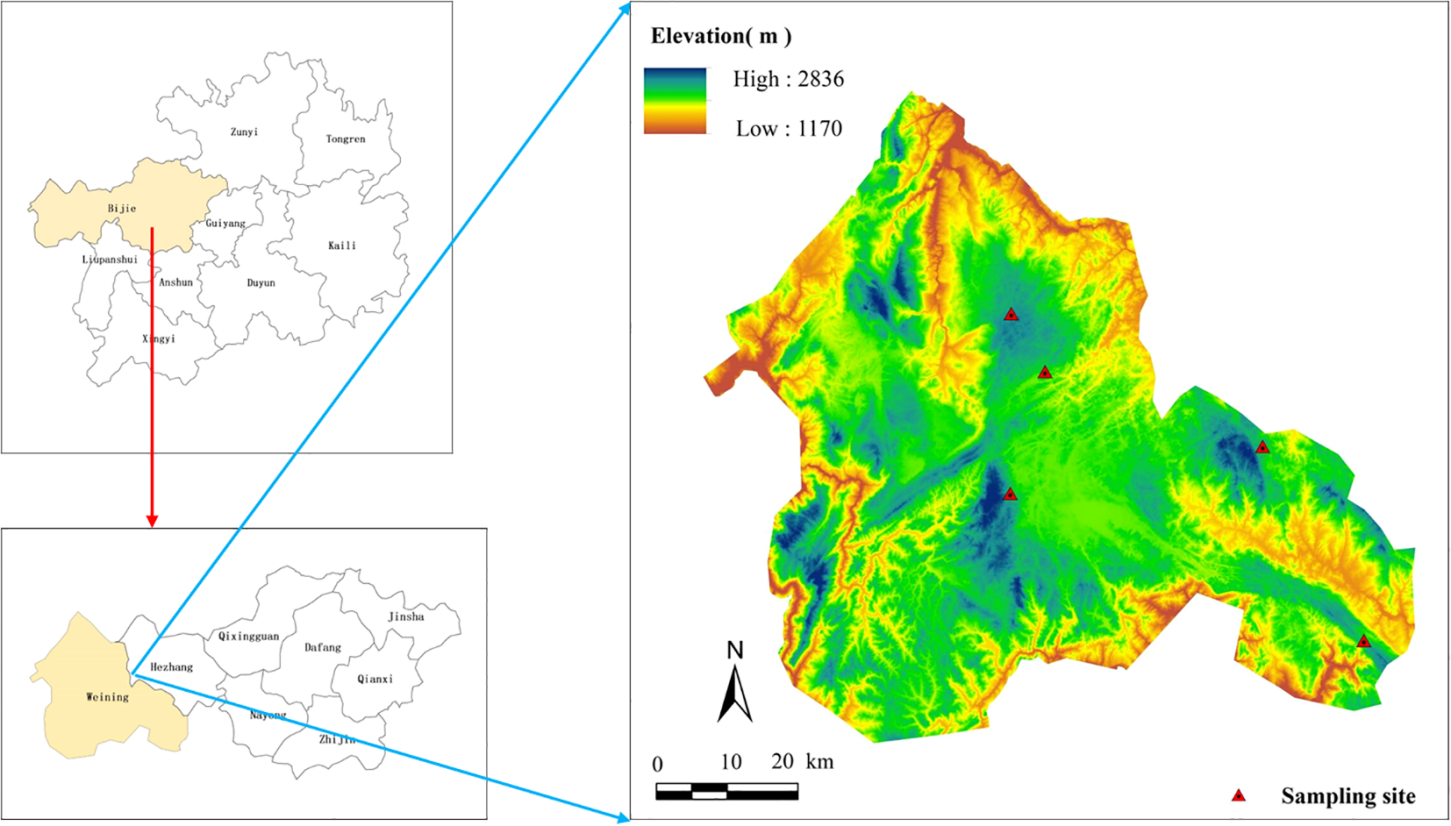
Distribution of sampling point of *P. armandii* in Bijie City, Guizhou Province, China.

The study was conducted in Bijie City(103°36′–106°43′ E, 26°21′–27°46′ N), located in Guizhou Province, China (**Figure 1**). The region lies within the core area of the contiguous karst zone of Yunnan-Guizhou-Guangxi, which belongs to the East Asian karst region-one of the three major global karst concentration areas. It is characterized by a subtropical humid monsoon climate with synchronous heat and moisture availability. Due to significant altitudinal variation, vertical climatic zonation is pronounced. The mean annual temperature ranges from 10 to 15°C, annual sunshine duration ranges from 1096 to 1769 hours, and the frost-free period lasts from 245 to 290 days. The dominant soil types include yellow soil and yellow-brown soil, with sporadic distributions of calcareous soil, purple soil, and paddy soil, exhibiting clear vertical zonation. All experimental sites in this study were situated on yellow-brown soil. The original vegetation was mid-subtropical evergreen broad-leaved forest. However, due to long-term human disturbance, primary forests are poorly preserved. The current vegetation is predominantly dominated by secondary and artificial forests. Common tree species include *P. armandii*, *Pinus yunnanensis*, *Pinus massoniana*, as well as species from the *Fagaceae* family (e.g., *Castanopsis* and *Cyclobalanopsis*) and the *Lauraceae* family (e.g., *Cinnamomum* and *Machilus*).

### 2.2 Sampling design

From September to October 2024, a transect was established along an elevational gradient within the main distribution area of *P. armandii* in Bijie City. This sampling period was strategically chosen to coincide with the late growing season, when leaf functional traits are relatively stable and plants undergo nutrient resorption for dormancy preparation. This timing was ideal for investigating altitudinal variations in leaf traits during a key phenological stage, thereby ensuring reliable comparisons across elevations. Five elevation levels were selected at 100 m intervals between 2100 and 2600 m (specifically: 2128 m, 2204 m, 2310 m, 2426 m, and 2509 m) (**Figure 1**). At each elevation, three 20 m×20 m quadrats were randomly established, resulting in a total of 15 quadrats. Within each quadrat, stand characteristics were recorded, including latitude, longitude, elevation, aspect, and slope.

In each quadrat, five healthy *P. armandii* individuals were selected as standard trees. From the middle outer part of the canopy, current-year branches were collected from the east, south, west, and north directions. From each branch, 50 bundles of healthy, undamaged needles were selected, wrapped in moist filter paper, and stored in a cooling box. Within each quadrat, three sampling points were randomly set up. At each sampling point, soil was collected from a depth of 0–20 cm using the five-point sampling method (centered on the point within a 1 m × 1 m area). After removing the surface litter, soil augers were used for sampling. The five soil subsamples from the same sampling point were mixed to form one composite sample. Thus, each quadrat yielded three independent composite samples, giving a total of 45 independent soil samples (15 quadrats × 3 samples per quadrat). All samples were sealed and stored for subsequent analysis.

### 2.3 Measurement of leaf functional traits and soil properties

LFTs were measured following the protocols described in the”Leaf traits”section of the Handbook for Standardized Measurement of Plant Functional Traits Worldwide (Pérez-Harguindeguy et al., 2013). A total of 12 LFTs, associated with plant growth and metabolic strategies, were assessed: Leaf Length (LL, cm), Leaf Thickness (LT, mm), Leaf Area (LA, cm^2^), Specific Leaf Area (SLA, cm^2^·g^−1^), Leaf Dry Matter Content (LDMC, mg·g^−1^), Leaf Carbon Content (LCC, g·kg^−1^), Leaf Nitrogen Content (LNC, g·kg^−1^), Leaf Phosphorus Content (LPC, g·kg^−1^), Leaf Potassium Content (LKC, g·kg^−1^), Leaf Carbon to Nitrogen Ratio (L_C:N_), Leaf Carbon to Phosphorus Ratio (L_C:P_), and Leaf Nitrogen to Phosphorus Ratio (L_N:P_).

Prior to measurement, needles were stored in darkness at 5°C for 12 hours, then surface-dried and weighed to determine leaf fresh weight (LFW) using an electronic balance with a precision of 0.001 g. Leaf images were acquired with a flatbed scanner, and LA was quantified using ImageJ software. LT was measured at three locations (base, middle, and apex) with a vernier caliper (precision: 0.02 mm), and the mean value was recorded. Subsequently, leaves were oven-dried at 105°C to deactivate enzymes, followed by drying at 80°C to constant weight to determine leaf dry weight (LDW). Dried leaf samples were ground and sieved for subsequent elemental analyses.

LNC and LPC was determined using a SEAL AA3 Continuous Flow Analyzer (Germany). LKC was measured with a Puxi A3 AFG-12 Flame Atomic Absorption Spectrophotometer (Wang et al., 2024). Similarly, soil N and P contents were analyzed with the continuous flow analyzer, and soil K was assessed via flame atomic absorption spectrometry. Soil pH was measured using the glass electrode method (Wang et al., 2024).

SLA and LDMC were calculated as follows:


$$SLA=\frac{LA}{LDW}$$

$$LDMC=\frac{LDW}{LFW}$$

### 2.4 Data analysis

All statistical analyses were performed in R 3.5.1. Spatial autocorrelation in trait data was assessed using Moran’s I test in the spdep package. A spatial weight matrix was constructed based on the inverse distance between the geographic coordinates of the 15 plots. The Moran’s I test revealed no significant spatial autocorrelation in any of the measured leaf functional traits (all |Moran’s I|< 0.1, *p*>0.05). On this basis, linear regression was considered appropriate for subsequent analysis. Linear regression models were employed to evaluate the relationships between elevation and leaf functional traits. Differences in leaf traits across elevation gradients were compared using one-way analysis of variance (ANOVA) followed by Tukey’s honestly significant difference (HSD) test for *post-hoc* comparisons. Pearson correlation and functional trait network were used to analyze trait correlations. Forward selection with double-stopping criteria identified key environmental factors, and redundancy analysis (RDA) explored trait-environment relationships. Additionally, path analysis was performed to partition the direct and indirect effects of environmental factors on traits, thereby elucidating the underlying mechanisms. All analyses were carried out using the vegan, car, agricolae, and spdep packages in R.

#### 2.4.1 Plant trait network construction

To investigate the co-variation relationships among plant traits, we constructed a plant trait network following these specific steps:

Data Preprocessing: All plant trait data were tested for normality using the Shapiro-Wilk test. The results indicated that most traits did not follow a normal distribution (*p* < 0.05). Therefore, we used the non-parametric Spearman’s rank correlation to calculate correlations between all trait pairs, as this method is insensitive to outliers and produces more robust results.

Correlation Matrix and Significance Testing: A matrix of Spearman’s correlation coefficients (r^s^) and their corresponding *p*-values was computed for all pairwise trait combinations.

Control of Spurious Correlations and Edge Filtering: To mitigate spurious correlations arising from multiple comparisons, we employed the False Discovery Rate (FDR) control method to correct the *p*-values (Benjamini, 2010). Specifically, the Benjamini-Hochberg procedure was used to calculate the FDR-adjusted q-value (which represents the minimum FDR at which a test may be called significant) for each correlation. Only correlations with q-value< 0.05 were considered statistically significant and retained as edges in the final network. This step is crucial for controlling the false positive rate and ensuring that network edges represent reliable ecological relationships.

Network Visualization and Analysis: The filtered matrix of significant correlations was imported into the igraph package for network visualization and analysis. In the resulting network, nodes represent plant traits, and edges represent Spearman correlations that remained significant after FDR correction. Edge thickness is proportional to the absolute value of the correlation coefficient, providing visual representation of association strength.

#### 2.4.2 Path analysis and variable selection

Path analysis was conducted to disentangle the direct and indirect effects of environmental factors on plant functional traits. The variable selection procedure was implemented as follows:

Initial Full Model Construction: For each plant trait, a full path model was constructed incorporating all eight soil factors.

Stepwise Variable Selection:

To mitigate multicollinearity, we computed the variance inflation factor (VIF) for each soil factor. Predictors with VIF values exceeding 10 - indicating severe multicollinearity - were sequentially excluded to ensure model stability and interpretability (James et al., 2013).

After addressing multicollinearity, a backward stepwise selection procedure was applied based on the Akaike information criterion (AIC). Predictors with the highest *p*-values were iteratively removed until all remaining path coefficients were statistically significant (*p* < 0.05).

Final Model Validation: The final path model for each trait retained only those variables that were both statistically significant and ecologically interpretable, ensuring a parsimonious and meaningful representation of the underlying processes.

## 3 Results

### 3.1 LFTs variation characteristics

Variance analysis revealed significant differences (*p<0.05*) in most LFTs across the elevational gradient, with the exception of L_N:P_ (**Table 1**). Specifically, LL, LT, SLA, and LDMC did not show significant differences among middle elevation gradients, but exhibited significant variation across other elevation ranges (*p<0.05*). In contrast, LA showed significant differences (*p<0.05*) across all five distinct elevation gradients. Significant differences (*p<0.05*) in LCC, LNC, LPC, LKC, as well as L_C:N_ and L_C:P_ were primarily observed between the highest elevation (2509 m) and the other gradients, indicating that these functional traits exhibit the greatest divergence between the highest elevation and other gradients.

**Table 1:** Leaf functional trait parameters and their coefficients of variation for *P. armandii* at different altitudinal gradients.

Leaf functional trait	2128 m	2204 m	2310 m	2426 m	2509 m	CV/%
LL/cm	14.97 ± 0.59a	13.80 ± 0.41b	13.17 ± 0.61bc	12.42 ± 0.59cd	12.13 ± 0.55d	8.67
LT/mm	0.87 ± 0.06a	0.78 ± 0.03b	0.74 ± 0.01bc	0.69 ± 0.01cd	0.66 ± 0.02d	10.56
LA/cm^2^	1.66 ± 0.01a	1.45 ± 0.08b	1.30 ± 0.06c	1.16 ± 0.06d	1.05 ± 0.05e	17.25
SLA/(cm^2^·g^−1^)	85.58 ± 0.48a	79.57 ± 3.39b	71.90 ± 0.58c	68.41 ± 2.47c	60.47 ± 5.35d	12.83
LDMC/(mg·g^−1^)	0.36 ± 0.02d	0.40 ± 0.01c	0.43 ± 0.01c	0.47 ± 0.02b	0.52 ± 0.03a	13.56
LCC/(g·kg^−1^)	501.02 ± 15.31ab	504.85 ± 4.38ab	506.68 ± 20.80a	484.75 ± 5.42ab	480.42 ± 14.35b	3.24
LNC/(g·kg^−1^)	11.38 ± 1.29b	12.92 ± 0.61b	12.57 ± 0.22b	12.22 ± 0.54b	15.00 ± 1.92a	12.15
LPC/(g·kg^−1^)	0.97 ± 0.09b	1.17 ± 0.34b	1.21 ± 0.24ab	1.42 ± 0.40ab	1.73 ± 0.25a	28.15
LKC/(g·kg^−1^)	5.96 ± 1.06a	5.59 ± 0.47a	5.59 ± 0.40a	4.97 ± 0.45a	3.49 ± 0.45b	20.42
L_C:N_	44.33 ± 4.15a	39.14 ± 2.23a	40.29 ± 1.01a	39.74 ± 2.09a	32.33 ± 3.63b	11.95
L_C:P_	519.48 ± 44.25a	458.17 ± 129.33ab	430.94 ± 89.55abc	357.51 ± 91.74bc	281.89 ± 45.20c	27.38
L_N:P_	11.73 ± 0.53a	11.68 ± 3.07a	10.71 ± 2.29a	9.02 ± 2.32a	8.87 ± 2.13a	21.96

LL, leaf length; LT, leaf thickness; LA, leaf area; SLA, specific leaf area; LDMC, leaf dry matter content; LCC, leaf carbon content; LNC, leaf nitrogen content; LPC, leaf phosphorus content; LKC, leaf potassium content; L_C:N_, leaf C/N ratio; L_C:P_, leaf C/P ratio; L_N:P_, leaf N/P ratio. Different lowercase letters within the same line indicate significant differences between altitudinal gradients at *p<0.05*.

The coefficients of variation (CV) for the LFTs of *P. armandii* ranged from 3.24% to 28.15%. Among these traits, LCC displayed the lowest variability (CV = 3.24%), suggesting minimal influence of elevational change on carbon allocation and relatively stable carbon metabolism. In contrast, LPC exhibited the highest variability (CV = 28.15%), reflecting a high sensitivity of phosphorus metabolism to the environmental gradients associated with elevation.

### 3.2 Altitudinal divergence in LFTs

**Figure 2:**
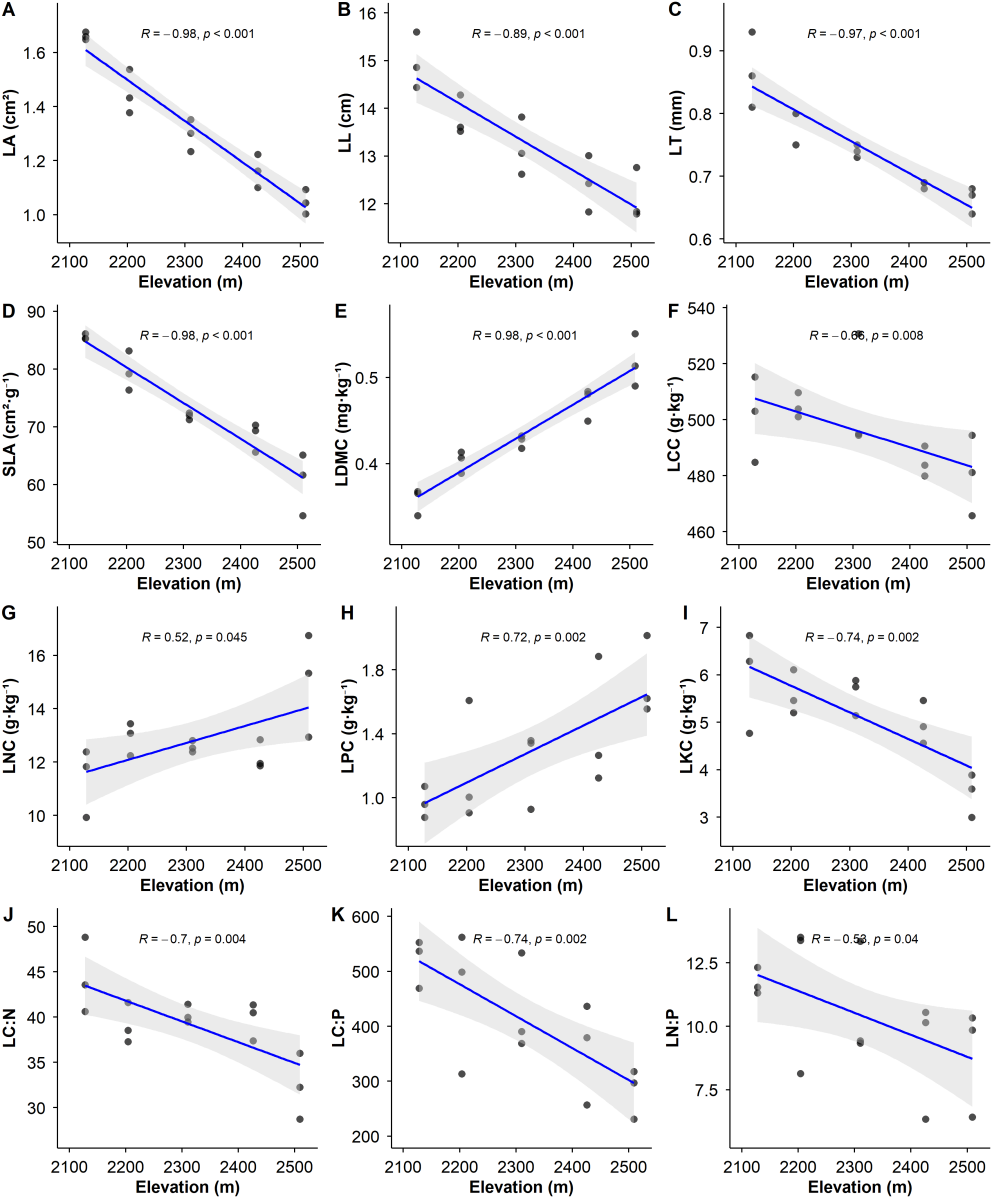
Trends in leaf functional trait parameters of *P. armandii* along the elevation gradient. In the figure, the regression line fitted by the solid line indicates a significant linear relationship (*p<0.05*), while the regression line fitted by the dashed line indicates a non-significant linear relationship (*p> 0.05*). The gray shaded area represents the 95% confidence interval.

Linear regression analysis revealed significant altitudinal divergence patterns in LFTs (**Figure 2**). SLA, LL, LT, and LA exhibited significant decreasing trends with increasing elevation (*p<0.01*). Conversely, LDMC showed a significant increasing trend (*p<0.01*). These morphological traits demonstrated strong correlations with elevation, which were either positive (*R>0.80*) or negative (*R<-0.80*).

LCC, LKC, L_C:N_, L_C:P_, and L_N:P_ decreased significantly with increasing elevation (*p<0.05*). In contrast, LNC and LPC increased significantly (*p<0.05*). These nutrient-related traits showed substantial correlations with elevation, which were either positive (*R>0.50*) or negative (*R<-0.50*). Notably, the strength of correlation between elevation and morphological traits (LL, LT, LA, SLA, LDMC) was substantially greater than those observed for nutrient traits (LCC, LKC, LNC, LPC, L_C:N_, L_C:P,_ L_N:P_).

### 3.3 Analysis of correlations among LFTs

**Figure 3:**
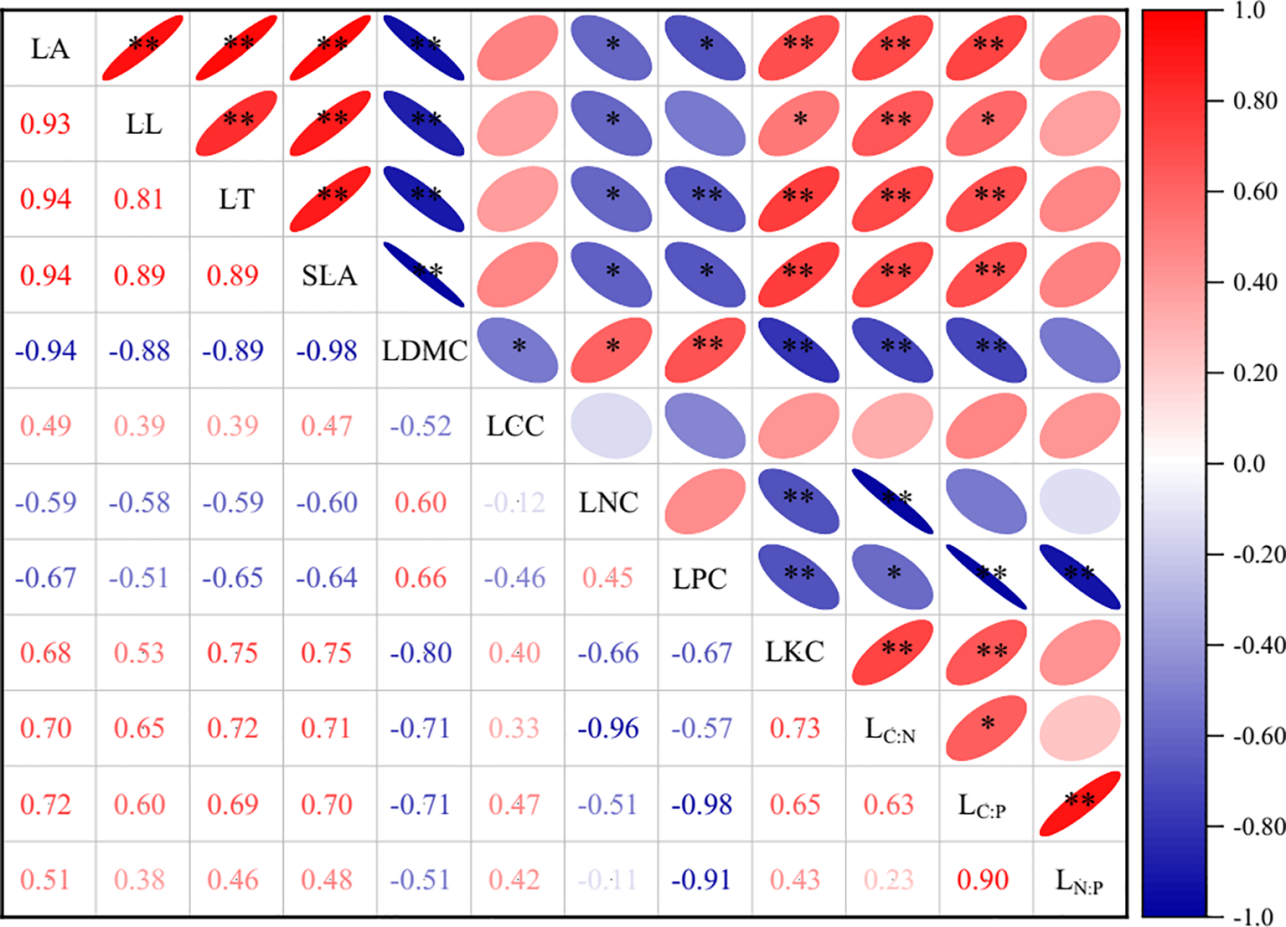
The correlation between leaf functional traits of *P. armandii*. Different numbers and color blocks represent Pearson correlation coefficients between different functional traits, with asterisks indicating the level of correlation significance (*p<0.05; **p<0.01).

Correlation analysis revealed widespread but variable significant relationships among LFTs of *P. armandii* (**Figure 3**). Notably, LDMC exhibited significant correlations with all traits except L_N:P_. LT, LA, SLA, and LKC were significantly associated with most traits, excluding LCC and L_N:P_. LL and LPC also showed strong connectivity, lacking significant correlations only with LCC, LPC, L_N:P_ and with LL, LCC, LNC, respectively. LNC correlated significantly with the majority of traits, showing non-significance only with LCC, LPC, L_C:P_, and L_N:P_. Conversely, LCC displayed the weakest connectivity, correlating significantly only with LDMC, while L_N:P_ exhibited limited connectivity, correlating significantly only with LPC and L_C:P_.

**Figure 4:**
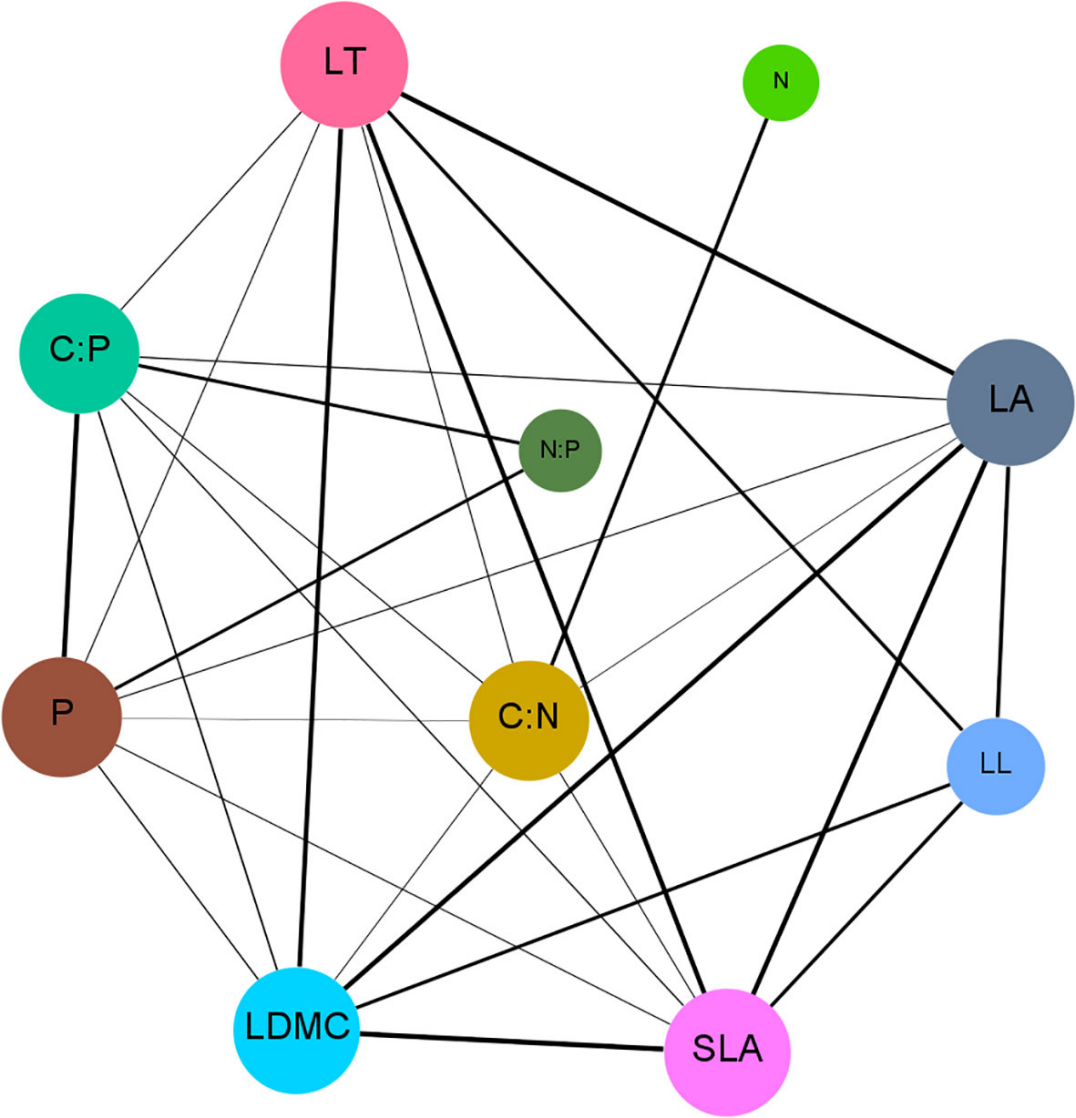
The correlation network among the functional traits of *P. armandii*. Each different node represents a distinct trait of the plant, and the edges represent the relationships between the traits.

The functional trait network constructed based on these correlations (**Figure 4**) further demonstrated that the LFTs of *P. armandii* form a tightly interconnected and highly coordinated complex network. Topological analysis of the network (**Table 2**) identified L_C:N_ as the most central hub trait, exhibiting the highest degree centrality (Degree=7), closeness centrality (Closeness=0.818), and betweenness centrality (Betweenness=0.222). This centrality highlighted the pivotal regulatory role of L_C:N_ in the environmental adaptation strategies of *P. armandii*.

**Table 2:** Node parameters of correlation network among leaf functional traits of *P. armandii*.

Leaf functional trait	Network node parameter			
	Degree	Closeness	Betweenness	Clustering coefficient
LA	7	0.818	0.035	0.857
LL	4	0.563	0.000	1.000
LT	7	0.818	0.035	0.857
SLA	7	0.818	0.035	0.857
LDMC	7	0.818	0.035	0.857
N	1	0.474	0.000	0.000
P	7	0.818	0.097	0.762
C:N	7	0.818	0.222	0.714
C:P	7	0.818	0.097	0.762
N:P	2	0.500	0.000	1.000

### 3.4 Response of LFTs to environmental factors

**Figure 5:**
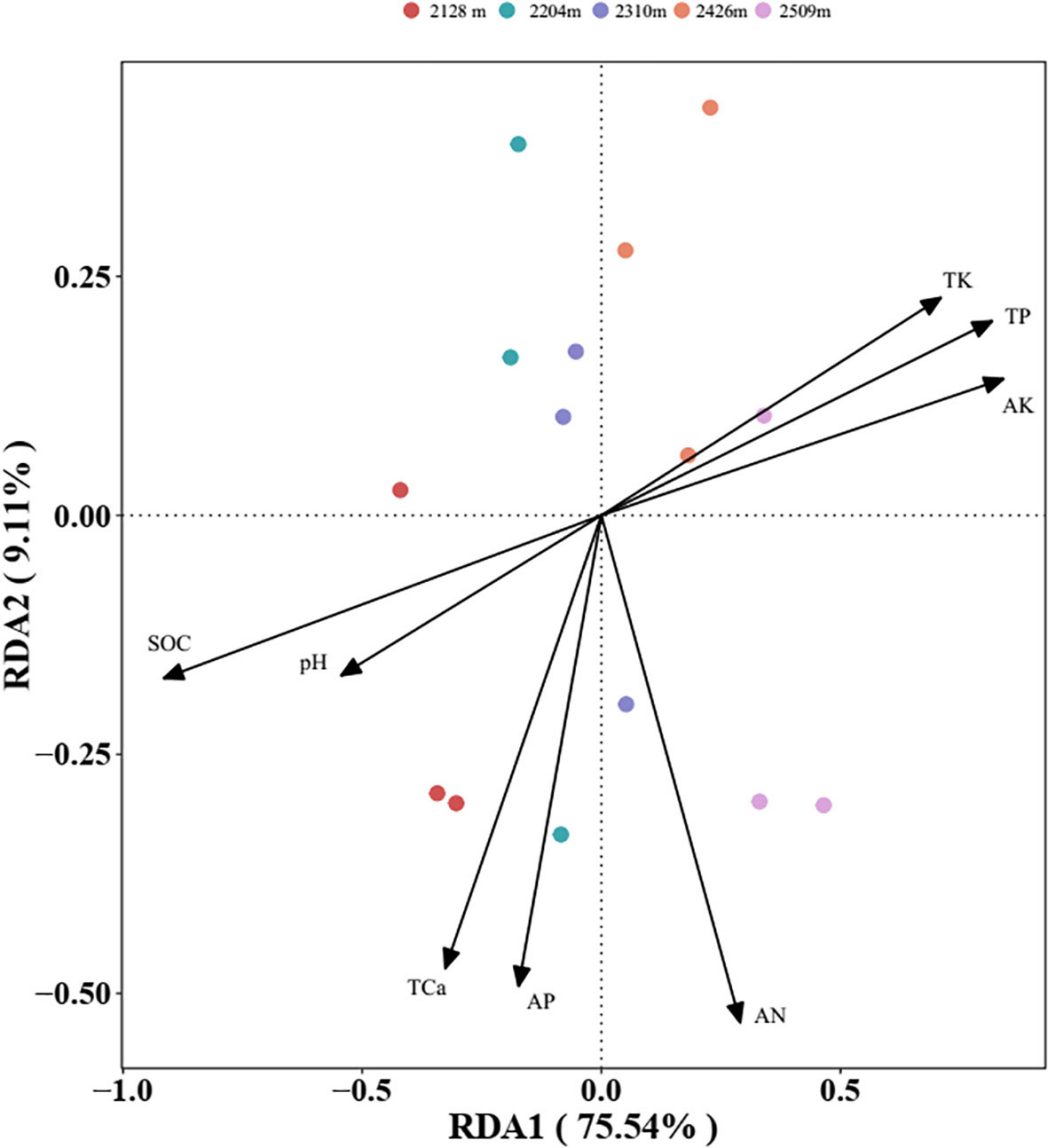
Redundancy analysis between the leaf functional traits of *P. armandii*. and soil factors.

Given the limited spatial scale of the study area and the minimal variation in climatic factors within the region, we hypothesized that soil properties would exert a greater influence on the variation in LFTs of *P. armandii*. Redundancy analysis (RDA) identified eight key soil variables: SOC, TP, TCa, TK, AN, pH, AK, and AP (**Figure 5**). Collectively, these factors explained 84.65% of the total variation observed in the LFTs. Among them, SOC, pH, and AK exhibited statistically significant correlations with the leaf functional traits (*p<0.05*), indicating their predominant role in driving the variation of leaf functional characteristics in *P. armandii*.

Path analysis of LFTs and environmental factors (**Table 3**) revealed significant divergence in the dominant environmental drivers among different leaf traits of *P. armandii*. Specifically, LL was primarily regulated by SOC, AP, and AK, with SOC and AP exerting positive effects, while AK exerted a negative effect. LT was mainly influenced by AP and AK; AK exerted a direct negative effect, while AP contributed a positive effect primarily through a significant indirect pathway. LA was predominantly regulated by SOC, pH, and AK, with SOC and pH exerting positive effects and AK exerting a negative effect. SLA was primarily governed by SOC, TCa, and TK, where SOC and TCa exerted positive effects and TK a negative effect. LDMC was mainly regulated by SOC, pH, AK, and AP; AK exerted a positive direct effect, SOC and pH exerted negative direct effects, and AP exerted a negative effect via a significant indirect pathway. LNC was predominantly influenced by TP, TCa, and pH; TP exerted a positive direct effect, whereas TCa and pH exerted negative direct effects. LKC was primarily driven by pH, AK, and AP, with pH exerting a positive direct effect and both AK and AP exerting negative direct effects. L_N:P_ was mainly controlled by TCa, pH, AK, and AP; pH exerted a positive direct effect, while TCa, AK, and AP exerted negative direct effects. L_C:P_ was predominantly influenced by pH and AK, with pH exerting a positive direct effect and AK exerting a negative direct effect. Additionally, pH exerted a direct positive effect on L_C:N_.

**Table 3:** Path coefficients of main influencing factors of leaf functional traits of *P. armandii*.

Leaf functional trait	Influencing factor	Correlation coefficient	Direct path coefficient	Total indirect path coefficient
LL/cm	SOC	0.9502	0.8497	0.1005
	AP	0.3910	0.2606	0.1303
	AK	-0.8014	-0.2280	-0.5734
LT/mm	AP	0.3205	-0.2215	0.5421
	AK	-0.7107	-0.5863	-0.1244
LA/cm^2^	SOC	0.9143	0.5466	0.3677
	pH	0.5804	0.3429	0.2374
	AK	-0.8101	-0.5309	-0.2792
SLA/ cm^2^·g^-1^	SOC	0.9253	0.5935	0.3318
	TCa	0.3820	0.1572	0.2247
	TK	-0.7891	-0.3179	-0.4712
LDMC/ mg·g-^1^	SOC	-0.9268	-0.5344	-0.3924
	pH	-0.5125	-0.4715	-0.0410
	AK	0.7634	0.3144	0.4489
	AP	-0.0791	0.3379	-0.4171
LNC/ g·kg^−1^	TP	0.5584	0.8025	-0.2441
	TCa	-0.2095	-0.4629	0.2533
	pH	-0.2727	-0.8366	0.5638
LKC/ g·kg^−1^	pH	0.2881	0.9305	-0.6424
	AK	-0.6881	-0.7650	0.0769
	AP	-0.2267	-0.7851	0.5584
L_C:N_	pH	0.3902	0.9760	-0.5857
L_C:P_	pH	0.4063	0.6959	-0.2895
	AK	-0.6063	-0.7225	0.1161
L_N:P_	TCa	-0.1620	-0.5207	0.3586
	pH	0.2709	0.7093	-0.4383
	AK	-0.3854	-0.5566	0.1711
	AP	-0.1148	-0.6792	0.5644

## 4 Discussion

As a primary organ for material accumulation and energy exchange, the leaf plays a fundamental role in ecosystem primary productivity. Leaf traits reflect key aspects of plant adaptation strategies developed through long-term evolution (Wright et al., 2005). Investigating how these traits vary along environmental gradients provides critical insights into the mechanisms underlying plant environmental adaptation.

### 4.1 Altitudinal effects on LFTs of *P. armandii*

Variation in altitude drives gradient changes in environmental factors such as temperature, precipitation, light availability, and soil properties, leading to pronounced redistribution of heat and moisture within relatively confined spatial scales. In response to these complex environmental shifts, plants exhibit adaptive adjustments in functional traits. This study revealed significant altitudinal variation (*p<0.05*) in LFTs of *P. armandii*, with coefficients of variation ranging from 3.24% to 28.15%, indicating considerable trait plasticity. These results suggest that *P. armandii* employs coordinated changes in multiple leaf functional traits to adapt to varying altitudinal habitats. While interspecific variation has traditionally been emphasized in plant functional trait studies, growing evidence indicates that intraspecific variation is also substantial, accounting for 28%-52% of total trait variation (Albert et al., 2010; Auger and Shipley, 2013; Jiang et al., 2016). In this study, the average intraspecific variation in LFTs of *P. armandii* was 16.80%, which is relatively low compared to other vegetation types. We propose several non-exclusive explanations for this constrained variation. First, it may indeed reflect limited morphological plasticity in response to the strong filtering effect of the harsh, high-elevation environmental conditions (Auger and Shipley, 2013), which selects for a narrow range of trait values. Second, the studied elevational gradient, while capturing the species’ primary distribution, might not encompass the full environmental spectrum necessary to elicit broader trait variation. Finally, as a long-lived conifer, *P. armandii* may possess an inherently conservative growth strategy, leading to lower intraspecific variability compared to more opportunistic herbaceous species commonly reported in other studies.

Plants adapt to environmental heterogeneity through functional traits such as LA, SLA, LDMC, LL, and LT. These traits collectively reflect strategies of resource acquisition and conservation (Yao et al., 2023). SLA, in particular, is a key structural trait related to light interception efficiency and environmental acclimation (Xu et al., 2023). In this study, SLA decreased significantly with increasing altitude, consistent with observations across multiple mountain ecosystems (Pensa et al., 2009; Rixen et al., 2022). This pattern can be explained by divergent resource-use strategies: at lower altitudes, where conditions are warmer and resources more abundant, plants prioritize light capture and photosynthetic rates by producing larger, thinner leaves with high SLA. At higher altitudes, plants invest in denser mesophyll tissues, reduce LA, and decrease SLA to limit respiration and transpiration, thereby enhancing photoprotection and adaptation to resource-limited environments (Gao and Liu, 2018; Liu et al., 2023a). This shift toward a conservative strategy aligns with observations that plants in arid or cold environments often prioritize structural durability over rapid growth (Melis et al., 2023; Poggiato et al., 2023).

Conversely, LDMC showed a significant positive correlation with altitude (*p<0.01*). LDMC reflects nutrient retention capacity and is largely independent of other leaf traits. Higher LDMC at elevated altitudes suggests greater investment in carbon-rich structural tissues, which reduces water loss by increasing diffusion resistance and enhances mechanical support under environmental stress. These findings align with reports that plants in high-altitude environments-characterized by low temperatures, high radiation, and nutrient-poor soils-allocate more biomass to supportive tissues, resulting in elevated LDMC (Saura-Mas and Lloret, 2007). A negative correlation between SLA and LDMC further supports a trade-off between resource acquisition and conservation strategies along the altitudinal gradient. However, the ecological implications of this relationship may vary between species due to divergent adaptive responses to altitude.

Carbon (C), nitrogen (N), and phosphorus (P) are major constituents of leaf dry mass and play vital roles in photosynthesis, cell growth, and differentiation (He et al., 2006). Their stoichiometric ratios provide valuable insights into plant metabolic strategies and environmental adaptability (Pulido et al., 2023). In this study, both LN and LP increased with elevation, consistent with results from a 3000-m elevation gradient on Gongga Mountain (Xu et al., 2021). This trend supports the Temperature-Plant Physiology Hypothesis (TPPH), which posits that low temperatures at high altitudes reduce metabolic and phosphate cycling rates, prompting plants to accumulate N and P to maintain photosynthetic and enzyme activity (e.g., Rubisco synthesis). These adjustments help compensate for reduced physiological efficiency under cold conditions and facilitate adaptation to short growing seasons and high light intensity.

L_C:N_ and L_C:P_ decreased with increasing elevation, indicating lower nitrogen and phosphorus use efficiency in high-altitude populations. This likely results from constrained leaf development and reduced photosynthetic rates under cooler and drier conditions, limiting nutrient accumulation and utilization. These findings align with the observed reduction in SLA, suggesting greater resource allocation to structural defense at the expense of growth-related functions at higher elevations (Rauf et al., 2023). The N:P ratio serves as an indicator of nutrient limitation. Across all five elevations, L_N:P_ was below 14, suggesting that nitrogen is the primary limiting nutrient for *P. armandii* growth in the study area (Koerselman and Meuleman, 1996). Leaf stoichiometric traits exhibited varying degrees of altitudinal sensitivity: phosphorus content showed the highest coefficient of variation, indicating stronger phenotypic plasticity and faster response to elevation changes compared to nitrogen, carbon, and C:N ratios (Zhang et al., 2023). This highlights the role of P in mediating plant adaptation to altitudinal gradients.

### 4.2 Coupling and trade-offs among LFTs

In the process of plant environmental adaptation, leaf functional traits do not operate in isolation but form integrated suites through trade-offs and synergies, as captured by the leaf economics spectrum framework (Fajardo and Siefert, 2018; Monge-González et al., 2021; Wright et al., 2007). These trait combinations reflect fundamental ecological strategies in resource allocation. However, the strength and direction of these trait relationships can vary across study scales. A key uncertainty lies in understanding how the response of a given trait along elevational gradients is constrained by its correlations with other traits (Midolo et al., 2019). This study demonstrates widespread significant correlations among LFTs in *P. armandii*, indicating that leaf traits respond to environmental stress in a modular and coordinated manner. This reflects a strategy optimizing carbon investment and return, consistent with the “fast-slow”trade-off framework of the plant economics spectrum. SLA and LDMC, key indicators of ecological strategies, represent opposing ends of the resource acquisition-conservation spectrum (van Bodegom et al., 2014). Here, they exhibited a highly significant negative correlation, consistent with previous studies (Reich et al., 1991; Wilson et al., 1999; Wright and Cannon, 2001; Wei et al., 2011). In high-elevation environments characterized by low temperatures, low SLA and high LDMC help reduce water loss by increasing resistance to water diffusion and prolonging the diffusion pathway, thereby extending carbon payback time and allocating more resources to defense and structural support, which enhances survival under resource-limited conditions (Cornelissen et al., 2003). Additionally, a significant negative correlation was observed between LDMC and LT, suggesting that at high altitudes, *P. armandii* adopts a “slow investment-return” strategy characterized by high LDMC and low LT, improving tolerance to adverse conditions through increased dry matter content. In contrast, at low elevations, a”fast investment-return”strategy with low LDMC and high LT is favored. Meanwhile, the positive correlation between SLA and LT may be related to leaf size effects on boundary layer dynamics and thermal regulation (Wright et al., 2017). Larger leaves with thicker boundary layers can reduce sensible heat exchange, influencing leaf temperature and transpiration rates, thereby aiding water conservation and reducing transpiration (Leigh et al., 2017).

Significant trade-offs were also observed between structural and chemical traits. LDMC was positively correlated with LNC and negatively with L_C:N_, indicating that higher nitrogen investment may support denser structural tissue synthesis and improve nitrogen use efficiency. Conversely, SLA was positively correlated with L_C:N_ and negatively with LNC, aligning with the leaf economics spectrum: high-SLA leaves favor rapid resource acquisition, whereas low-SLA leaves reflect a conservative strategy (Wright et al., 2004). This coordinated variation between structural and chemical traits reflects an adaptive mechanism through which plants optimize resource use efficiency by balancing photosynthetic gain, structural investment, and metabolic cost in nutrient-poor habitats, consistent with the role of trait coordination in ecosystem functional recovery emphasized by Gao et al. (2025). Furthermore, LCC was significantly correlated only with LDMC, suggesting that carbon accumulation is primarily driven by structural demands rather than metabolic requirements. L_N:P_ was correlated only with LPC and L_C:P_, indicating that nitrogen and phosphorus allocation may be independently regulated by genetic constraints or soil phosphorus availability. The lack of a significant correlation between LNC and LPC along the elevational gradient further suggests that nutrient limitations (e.g., low temperature or soil nutrient availability) may decouple stoichiometric relationships, reflecting adaptive divergence of *P. armandii* across heterogeneous habitats.

In the trait network, traits with high node degree centrality often play core roles in functional integration (Flores-Moreno et al., 2019). In this study, L_C:N_ emerged as a central node (with the highest degree, closeness, and betweenness centrality), indicating that carbon-nitrogen balance plays a pivotal regulatory role in trait coordination. This finding supports the ecological stoichiometry theory, which posits that carbon-to-nitrogen ratios mediate growth-defense trade-offs. A high L_C:N_ typically signifies resource allocation to structural defense, whereas a low ratio promotes nitrogen metabolism and growth, suggesting that *P. armandii* dynamically adjusts carbon and nitrogen allocation to balance photosynthetic, structural, and defensive investments, thereby enhancing environmental adaptability.

### 4.3 Environmental drivers of LFTs

It is well-established that plant leaf functional traits are influenced by a range of abiotic and biotic factors, including climate (Sardans and Penuelas, 2014; Yuan and Chen, 2009), soil properties (Chen et al., 2013; Zhang et al., 2019), and plant life forms (He et al., 2006; Zhao et al., 2018). At broad spatial scales, climatic variables are often the dominant drivers of trait variation (Craine and Lee, 2003), whereas at finer regional scales, soil factors become particularly influential (Luo et al., 2019). Soil conditions affect plant growth through both direct and indirect pathways, serving as a primary source of nutrients and mediating resource availability (Peguero et al., 2023; Chen et al., 2024). Variations in nutrient supply can significantly impact plant functional traits, thereby influencing plant performance (Zhang et al., 2023). In line with these observations, our study demonstrated that multiple soil nutrients collectively influence the LFTs of *P. armandii*, though distinct traits being influenced by different key factors.

First, soil organic carbon (SOC) exerted significant positive direct effects on multiple leaf traits, particularly LL, LA, and SLA. This indicates that SOC promotes rapid leaf expansion and enhances light capture capacity by improving soil structure and nutrient availability, aligning with a “resource-acquisitive” ecological strategy. Conversely, SOC showed a significant negative correlation with LDMC, further supporting the idea that plants in high-SOC environments may prioritize resource allocation toward growth rather than structural defense. These findings align with previous studies by Huang et al. (2018) and Duan et al. (2017), which identified SOC as a key driver of divergence in plant functional traits. Similarly, Fan et al. (2022) reported SOC as a critical soil factor influencing LFTs in *Pinus tabulaeformis*. SOC, derived mainly from the decomposition of plant, animal, and microbial residues, as well as litter input, significantly modulates plant functional trait variation. Furthermore, by improving soil structure and enhancing water and nutrient retention, SOC provides plants with more sufficient moisture and nutrients, thereby facilitating transpiration and photosynthesis and promoting plant growth and development (Kühn et al., 2021).

Second, soil pH also significantly influenced multiple traits, particularly stoichiometric traits such as L_C:N_ and L_C:P_, indicating that soil acidity or alkalinity plays a crucial role in regulating internal carbon, nitrogen, and phosphorus balance in plants. The underlying mechanism may involve pH-induced changes in microbial activity and nutrient availability, which indirectly modulate elemental stoichiometry in plants (Liu et al., 2023b). In high-pH environments, plants tended to accumulate more carbon, reflecting a conservative resource-use strategy likely driven by nutrient limitations (e.g., reduced phosphorus availability) under alkaline conditions (Lin et al., 2022).

Notably, available potassium (AK) was negatively correlated with most leaf traits, particularly LL, LT, LA, and LDMC. For instance, the direct path coefficients of AK on LA and SLA were -0.5309 and -0.3179, respectively, suggesting that potassium availability may inhibit leaf expansion and specific leaf area, thereby promoting a more conservative leaf construction strategy. Although potassium is essential for plant growth, excess levels may inhibit leaf development via ionic toxicity or antagonism with other elements (e.g., calcium, magnesium), while also increasing LDMC- indicating a potential shift toward higher tissue density to maintain water and ionic balance under potassium stress.

Moreover, available phosphorus (AP) exhibited a complex relationship with LT: although their correlation coefficient was positive (0.3205), the direct path coefficient was negative (-0.2215), while the indirect effect was positive (0.5421). This implies that AP may indirectly promote leaf thickening through interactions with other factors (e.g., SOC or AK), rather than exerting a direct positive effect. This indirect influence highlights the complex synergistic and antagonistic interactions among soil factors, underscoring the need for multi-path analysis rather than relying solely on simple correlations when interpreting the effects of phosphorus on plant morphogenesis. Consistent with our findings, Chen et al. (2024) also identified soil phosphorus as a key factor influencing leaf functional traits.

## 5 Conclusion

This study examined LFTs of *P. armandii* along a small-scale elevational gradient, revealing significant altitudinal variation and coordinated trait relationships indicative of ecological adaptation to local environmental conditions. Key leaf traits exhibited synergistic or trade-off relationships in response to shifts in hydrothermal factors and soil properties along the gradient. Multiple soil drivers influenced these traits, with SOC, pH, and AK identified as primary factors, although different traits were influenced by distinct environmental factors. These results illuminate how *P. armandii* in karst mountainous areas adjusts to elevation-dependent environmental conditions, highlighting mechanisms underlying plant adaptation to changing habitats. The findings further imply that through trait integration, plants achieve a balance between growth and survival, offering insights for ecological restoration in rocky desertification zones. Future research should incorporate broader environmental dimensions-such as climate and topography-and extend trait analysis across multiple plant organs and functional groups to improve multi-scale understanding of trait-environment-ecosystem linkages. Particularly critical is exploring how global change alters soil nutrient availability and subsequently affects plant traits and ecosystem functioning.

## Data Availability

The original contributions presented in the study are included in the article/supplementary material. Further inquiries can be directed to the corresponding authors.

## References

[B1] Ackerly D. D. Cornwell W. K. (2007). A trait-based approach to community assembly: partitioning of species trait values into within-and among-community components. Ecol. Lett. 10, 135–145. doi: 10.1111/j.1461-0248.2006.01006.x, PMID: 17257101

[B2] Akram M. A. Wang X. Shrestha N. Zhang Y. Sun Y. Yao S. . (2023). Variations and driving factors of leaf functional traits in the dominant desert plant species along an environmental gradient in the drylands of China. Sci. Total Environ. 897, 165394. doi: 10.1016/j.scitotenv.2023.165394, PMID: 37437630

[B3] Albert C. H. Thuiller W. Yaccoz N. G. Soudant A. Boucher F. Saccone P. . (2010). Intraspecific functional variability: extent, structure and sources of variation. J. Ecol. 98, 604–613. doi: 10.1111/j.1365-2745.2010.01651.x

[B4] Anderson J. T. Gezon Z. J. (2015). Plasticity in functional traits in the context of climate change: a case study of the subalpine forb *Boechera stricta* (Brassicaceae). Global Change Biol. 21, 1689–1703. doi: 10.1111/gcb.12770, PMID: 25470363

[B5] Auger S. Shipley B. (2013). Inter-specific and intra-specific trait variation along short environmental gradients in an old-growth temperate forest. J. Veg. Sci. 24, 419–428. doi: 10.1111/j.1654-1103.2012.01473.x

[B6] Batllori E. Lloret F. Aakala T. Anderegg W. R. L. Aynekulu E. Bendixsen D. P. . (2020). Forest and woodland replacement patterns following drought-related mortality. Proc. Natl. Acad. Sci. Unit. States Am. 117, 29720–29729. doi: 10.1073/pnas.2002314117, PMID: 33139533 PMC7703631

[B7] Benjamini Y. (2010). Discovering the false discovery rate. J. R. Stat. Soc B. 72, 405–416. doi: 10.1111/j.1467-9868.2010.00746.x

[B8] Callaway R. M. Brooker R. W. Choler P. Kikvidze Z. Lortie C. J. Michalet R. . (2002). Positive interactions among alpine plants increase with stress. Nature 417, 844. doi: 10.1038/nature00812, PMID: 12075350

[B9] Cardinale B. J. Duffy J. E. Gonzalez A. Hooper D. U. Perrings C. Venail P. . (2012). Biodiversity loss and its impact on humanity. Nature 486, 7401–7467. doi: 10.1038/nature11148, PMID: 22678280

[B10] Chapin F. S. Zavaleta E. S. Eviner V. T. Naylor R. L. Vitousek P. M. Reynolds H. L. . (2000). Consequences of changing biodiversity. Nature 405, 234–242. doi: 10.1038/35012241, PMID: 10821284

[B11] Chen Y. H. Han W. X. Tang L. Y. Tang Z. Y. Fang J. Y. (2013). Leaf nitrogen and phosphorus concentrations of woody plants differ in responses to climate, soil and plant growth form. Ecography 36, 178–184. doi: 10.1111/j.1600-0587.2011.06833.x

[B12] Chen Z. Li Q. Jiang Z. Yan P. Arif M. (2024). Leaf functional traits of Daphniphyllum macropodum across different altitudes in Mao’er Mountain in Southern China. Front. For. Glob. Change 7. doi: 10.3389/ffgc.2024.1359361

[B13] Chevin L. M. Lande R. (2010). When do adaptive plasticity and genetic evolution prevent extinction of a density-regulated population? Evolution 64, 1143–1150. doi: 10.1111/j.1558-5646.2009.00875.x, PMID: 19863583

[B14] Cornelissen J. H. C. Lavorel S. Garnier E. Díaz S. Buchmann N. Gurvich D. E. . (2003). A handbook of protocols for standardised and easy measurement of plant functional traits worldwide. Aust. J. Bot. 51, 335. doi: 10.1071/BT02124

[B15] Craine J. M. Lee W. G. (2003). Covariation in leaf and root traits for native and non-native grasses along an altitudinal gradient in New Zealand. Oecologia 134, 471–478. doi: 10.1007/s00442-002-1155-6, PMID: 12647118

[B16] De Deyn G. B. de Cornelissen J. H. C. Bardgett R. D. (2008). Plant functional traits and soil carbon sequestration in contrasting biomes. Ecol. Lett. 11, 516–531. doi: 10.1111/j.1461-0248.2008.01164.x, PMID: 18279352

[B17] De Laender F. Rohr J. R. Ashauer R. Baird D. J. Berger U. Eisenhauer N. . (2016). Reintroducing environmental change drivers in biodiversity–ecosystem functioning research. Trends Ecol. Evol. 31, 905–915. doi: 10.1016/j.tree.2016.09.007, PMID: 27742415 PMC5118049

[B18] Díaz S. Kattge J. Cornelissen J. H. Wright I. J. Lavorel S. Dray S. . (2016). The global spectrum of plant form and function. Nature 529, 167–171. doi: 10.1038/nature16489, PMID: 26700811

[B19] Dong Z. J. Guo Y. R. Hou L. (2022). Nutrition diagnosis and fertilization of Pinus armandii forest in the Qingling Mountains, China. Chin. J. Appl. Ecol. 33, 2051–2056. doi: 10.13287/j.1001-9332.202208.001, PMID: 36043810

[B20] Duan Y. Y. Song L. J. Niu S. Q. Huang T. Yang G. H. Hao W. F. (2017). Variation in leaf functional traits of different-aged Robinia pseudoacacia communities and relationships with soil nutrients. Chin. J. Appl. Ecol. 28, 28–36. doi: 10.13287/j.1001-9332.201701.036, PMID: 29749185

[B21] Fajardo A. Siefert A. (2018). Intraspecific trait variation and the leaf economics spectrum across resource gradients and levels of organization. Ecology 99, 1024–1030. doi: 10.1002/ecy.2194, PMID: 29603183

[B22] Fan B. Ma Z. Gao P. Lu J. Ding N. Sun K. (2022). Functional traits of male and female leaves of hippophae tibetana on the eastern edge of the tibetan plateau and their altitudinal variability. Plants 11, 2484. doi: 10.3390/plants11192484, PMID: 36235349 PMC9573225

[B23] Flores-Moreno H. Fazayeli F. Banerjee A. Datta A. Kattge J. Butler E. E. . (2019). Robustness of trait connections across environmental gradients and growth forms. Global Ecol. Biogeog. 28, 1806–1826. doi: 10.1111/geb.12996

[B24] Frey S. J. K. Hadley A. S. Johnson S. L. Schulze M. Jones J. A. Betts M. G. (2016). Spatial models reveal the microclimatic buffering capacity of old-growth forests. Sci. Adv. 2, e1501392. doi: 10.1126/sciadv.1501392, PMID: 27152339 PMC4846426

[B25] Fu G. Sun W. (2022). Temperature sensitivities of vegetation indices and aboveground biomass are primarily linked with warming magnitude in high-cold grasslands. Sci. Total Environ. 843, 157002. doi: 10.1016/j.scitotenv.2022.157002, PMID: 35772540

[B26] Gao J. Liu Y. (2018). Climate stability is more important than water-energy variables in shaping the elevational variation in species richness. Ecol. Evol. 8, 6872–6879. doi: 10.1002/ece3.4202, PMID: 30073051 PMC6065338

[B27] Gao Y. Liu J. Wang D. J. An Y. Ma H. Y. Tong S. Z. (2025). Synergy and trade-off between plant functional traits enhance grassland multifunctionality under grazing exclusion in a semi-arid region. J. Environ. Manage. 373, 123877. doi: 10.1016/j.jenvman.2024.123877, PMID: 39733684

[B28] Graae B. J. Frenne P. D. Kolb A. Brunet J. Chabrerie O. Verheyen K. . (2012). On the use of weather data in ecological studies along altitudinal and latitudinal gradients. Oikos 121, 3–19. doi: 10.1111/j.1600-0706.2011.19694.x

[B29] Han M. G. Chen Y. Sun L. J. Yu M. Li R. Li S. F. . (2023). Linking rhizosphere soil microbial activity and plant resource acquisition strategy. J. Ecol. 111, 875–888. doi: 10.1111/1365-2745.14067

[B30] He X. Arif M. Zheng J. Ni X. Yuan Z. Zhu Q. . (2023). Plant diversity patterns along an elevation gradient: The relative impact of environmental and spatial variation on plant diversity and assembly in arid and semi-arid regions. Front. Environ. Sci. 11. doi: 10.3389/fenvs.2023.1021157

[B31] He J. S. Fang J. Wang Z. Guo D. Flynn D. F. Geng Z. (2006). Stoichiometry and large-scale patterns of leaf carbon and nitrogen in the grassland biomes of China. Oecologia 149, 115–122. doi: 10.1111/1365-2745.14067 16639565

[B32] He N. Li Y. Liu C. Xu L. Li M. Zhang J. . (2020). Plant trait networks: Improved resolution of the dimensionality of adaptation. Trends Ecol. Evol. 35, 908–918. doi: 10.1016/j.tree.2020.06.003, PMID: 32595068

[B33] Huang X. Yao L. Wang J. Zhu Q. Wu M. L. Liu Y. (2018). Effect of soil nutrients on leaf functional traits of different life form plants. Acta Bot. Boreal. - Occident. Sin. 38, 2293–2302. doi: 10.7606/j.issn.1000-4025.2018.12.2293

[B34] James G. Witten D. Hastie T. Tibshirani R. (2013). An introduction to statistical learning: with applications in R (New York, NY: Springer).

[B35] Jiang Y. Zang R. Letcher S. G. Ding Y. Huang Y. Lu X. . (2016). Associations between plant composition/diversity and the abiotic environment across six vegetation types in a biodiversity hotspot of Hainan Island, China. Plant Soil 403, 21–35. doi: 10.1007/s11104-015-2723-y

[B36] Kattge J. Díaz S. Lavorel S. Prentice I. C. Leadley P. Bonisch G. . (2011). TRY-a global database of plant traits. Glob. Chang Biol. 17, 2905–2935. doi: 10.1111/j.1365-2486.2011.02451.x

[B37] Klich M. G. (2000). Leaf variations in Elaeagnus angustifolia related to environmental heterogeneity. Environ. Exp. Bot. 44, 171–183. doi: 10.1016/S0098-8472(00)00056-3, PMID: 11064038

[B38] Koerselman W. Meuleman A. F. M. (1996). The vegetation N: P ratio: a new tool to detect the nature of nutrient limitation. J. App. Ecol. 33, 1441–1450. doi: 10.2307/2404783

[B39] Körner C. (1991). Some often overlooked plant characteristics as determinants of plant growth: a reconsideration. Funct. Ecol. 5, 162–173. doi: 10.2307/2389254

[B40] Körner C. (2007). The use of ‘altitude’ in ecological research. Trends Ecol. Evolution. 22, 569–574. doi: 10.1016/j.tree.2007.09.006, PMID: 17988759

[B41] Kühn P. Ratier Backes A. Romermann C. Bruelheide H. Haider S. (2021). Contrasting patterns of intraspecific trait variability in native and non-native plant species along an elevational gradient on Tenerife, Canary Islands. Ann. Bot. 127, 565–576. doi: 10.1093/aob/mcaa067, PMID: 32318707 PMC7988510

[B42] Kumari M. Pradhan U. K. Joshi R. Punia A. Shankar R. Kumar R. (2021). In-depth assembly of organ and development dissected Picrorhiza kurroa proteome map using mass spectrometry. BMC Plant Biol. 21, 604. doi: 10.1186/s12870-021-03394-8, PMID: 34937558 PMC8693493

[B43] Leigh A. Sevanto S. Close J. D. Nicotra A. B. (2017). The influence of leaf size and shape on leaf thermal dynamics: Does theory hold up under natural conditions? Plant Cell Environ. 40, 237–248. doi: 10.1111/pce.12857, PMID: 28026874

[B44] Li J. X. Liu Y. M. Peng J. F. Lv R. S. He Z. X. Li J. K. . (2025). Stability assessment of response of *Pinus armandii Franch.* Radial growth to climate change at high altitude in Funiu Mountains, Central China. Acta Ecol. Sin. 45, 157–167. doi: 10.1007/s11676-024-01698-7

[B45] Lin Y. T. Lai Y. Tang S. B. Qin Z. F. Liu J. F. Kang F. F. . (2022). Climatic and edaphic variables determine leaf C, N, P stoichiometry of deciduous Quercus species. Plant Soil 474, 383–394. doi: 10.1007/s11104-022-05342-3

[B46] Liu C. Y. Jiang M. T. Yuan M. M. Wang E. T. Bai Y. Crowther T. W. . (2023b). Root microbiota confers rice resistance to aluminium toxicity and phosphorus deficiency in acidic soils. Nat. Food 4, 912–924. doi: 10.1038/s43016-023-00848-0, PMID: 37783790

[B47] Liu C. Li Y. Yan P. He N. (2021a). How to improve the predictions of plant functional traits on ecosystem functioning? Front. Plant Sci. 12. doi: 10.3389/fpls.2021.622260, PMID: 33633766 PMC7901955

[B48] Liu X. Shi X. Zhang S. (2021b). Soil abiotic properties and plant functional diversity coregulate the impacts of nitrogen addition on ecosystem multifunctionality in an alpine meadow. Sci. Total Environ. 780, 146476. doi: 10.1016/j.scitotenv.2021.146476, PMID: 33773353

[B49] Liu C. Wang X. Wu X. Dai S. He J.-S. Yin W. (2013). Relative effects of phylogeny, biological characters and environments on leaf traits in shrub biomes across central Inner Mongolia, China. J. Plant Ecol. 6, 220–231. doi: 10.1093/jpe/rts028

[B50] Liu Z. Zhao M. Zhang H. Ren T. Liu C. He N. (2023a). Divergent response and adaptation of specific leaf area to environmental change at different spatiotemporal scales jointly improve plant survival. Glob. Change Biol. 29, 1144–1159. doi: 10.1111/gcb.16518, PMID: 36349544

[B51] Luo Y. Hu H. Zhao M. Li H. Liu S. Fang J. (2019). Latitudinal pattern and the driving factors of leaf functional traits in 185 shrub species across eastern China. J. Plant Ecol. 12, 67–77. doi: 10.1093/jpe/rtx065

[B52] Martin R. E. Asner G. P. (2009). Leaf chemical and optical properties of Metrosideros polymorpha across environmental gradients in Hawaii. Biotropica 41, 292–301. doi: 10.1111/j.1744-7429.2009.00491.x

[B53] Melis R. Morillas L. Roales J. Costa-Saura J. M. Lo Cascio M. Spano D. . (2023). Functional traits related to competition for light influence tree diameter increments in a biodiversity manipulation experiment. Eur. J. For. Res. 142, 709–722. doi: 10.1007/s10342-023-01552-1

[B54] Midolo G. de Frenne P. Hölzel N. Wellstein C. (2019). Global patterns of intraspecific leaf trait responses to elevation. Global Change Biol. 25, 2485–2498. doi: 10.1111/gcb.14646, PMID: 31056841

[B55] Monge-González M. L. Guerrero-Ramírez N. Kromer T. Kreft H. Craven D. (2021). Functional diversity and redundancy of tropical forests shift with elevation and forest-use intensity. J. Appl. Ecol. 58, 1827–1837. doi: 10.1111/1365-2664.13955

[B56] Navas M. L. Roumet C. Bellmann A. Laurent G. Garnier E. (2010). Suites of plant traits in species from different stages of a Mediterranean secondary succession. Plant Biol. 12, 183–196. doi: 10.1111/j.1438-8677.2009.00208.x, PMID: 20653901

[B57] Ni X. Sun L. Cai Q. Ma S. Feng Y. Sun Y. . (2022). Variation and determinants of leaf anatomical traits from boreal to tropical forests in eastern China. Ecol. Indic. 140, 108992. doi: 10.1016/j.ecolind.2022.108992

[B58] Nicotra A. B. Beever E. A. Robertson A. L. Hofmann G. E. O’Leary J. (2015). Assessing the components of adaptive capacity to improve conservation and management efforts under global change. Conserv. Biol. 29, 1268–1278. doi: 10.1111/cobi.12522, PMID: 25926277

[B59] Onoda Y. Wright I. J. Evans J. R. Hikosaka K. Kitajima K. Niinemets Ü. . (2017). Physiological and structural tradeoffs underlying the leaf economics spectrum. New Phytol. 214, 1447–1463. doi: 10.1111/nph.14496, PMID: 28295374

[B60] Ordoñez J. C. Van Bodegom P. M. Witte J. P. M. Wright I. J. Reich P. B. Aerts R. (2009). A global study of relationships between leaf traits, climate and soil measures of nutrient fertility. Glob. Ecol. Biogeogr. 18, 137–149. doi: 10.1111/j.1466-8238.2008.00441.x

[B61] Pan F. J. Zhang W. Wang K. L. He X. Y. Liang S. C. Wei G. F. (2011). Litter C:N:P ecological stoichiometry character of plant communities in typical karst Peak-Cluster depression. Acta Ecol. Sin. 31, 335–343. doi: 10.20103/j.stxb.2011.02.005

[B62] Peguero G. Coello F. Sardans J. Asensio D. Grau O. Llusià J. . (2023). Nutrient-based species selection is a prevalent driver of community assembly and functional trait space in tropical forests. J. Ecol. 111, 1218–1230. doi: 10.1111/1365-2745.14089

[B63] Pensa M. Karu H. Luud A. Kund K. (2009). Within-species correlations in leaf traits of three boreal plant species along a latitudinal gradient. Plant Ecol. 208, 155–166. doi: 10.1007/s11258-009-9695-z

[B64] Pérez-Harguindeguy N. Díaz S. Garnier E. Lavorel S. Poorter H. Jaureguiberry P. . (2013). New handbook for standardised measurement of plant functional traits worldwide. Aust. J. Bot. 61, 167. doi: 10.1111/1365-2435.12776

[B65] Pfennigwerth A. A. Bailey J. K. Schweitzer J. A. (2017). Trait variation along elevation gradients in a dominant woody shrub is population specific and driven by plasticity. AoB Plants 9, plx027. doi: 10.1093/aobpla/plx027, PMID: 28721188 PMC5509947

[B66] Poggiato G. Gaüzere P. Martinez-Almoyna C. Deschamps G. Renaud J. Violle C. . (2023). Predicting combinations of community mean traits using joint modelling. Glob. Ecol. Biogeogr 32, 1409–1422. doi: 10.1111/geb.13706

[B67] Poorter H. Niinemets U. Poorter L. Wright I. J. Villar R. (2009). Causes and consequences of variation in leaf mass per area (LMA): A meta-analysis. New Phytol. 182, 565–588. doi: 10.1111/j.1469-8137.2009.02830.x, PMID: 19434804

[B68] Pulido C. F. Rasmussen J. Eriksen J. Abalos D. (2023). Cover crops for nitrogen loss reductions: Functional groups, species identity and traits. Plant Soil 507, 127–140. doi: 10.1007/s11104-023-05895-x

[B69] Rasmann S. Pellissier L. Defossez E. Jactel H. Kunstler G. (2014). Climate-driven change in plant-insect interactions along elevation gradients. Funct. Ecol. 28, 46–54. doi: 10.1111/1365-2435.12135

[B70] Rauf Z. Zarif N. Khan A. Siddiqui S. Fatima S. Iqbal W. . (2023). The Western Himalayan fir tree ring record of soil moisture in Pakistan since 1855. Int. J. Biometeorol. 67, 1477–1492. doi: 10.1007/s00484-023-02517-0, PMID: 37464201

[B71] Reich P. B. Uhl C. Walters M. B. Ellsworth D. S. (1991). Leaf lifespan as a determinant of leaf structure and function among 23 amazonian tree species. Oecologia 86, 16–24. doi: 10.1007/BF00317383, PMID: 28313152

[B72] Reich P. B. Wright I. J. Cavender-Bares J. Craine J. M. Oleksyn J. Westoby M. . (2003). The evolution of plant functional variation: traits, spectra, and strategies. Int. J. Plant Sci. 164, S143–S164. doi: 10.1086/374368

[B73] Rixen C. Wipf S. Rumpf S. B. Giejsztowt J. Millen J. Morgan J. W. . (2022). Intraspecific trait variation in alpine plants relates to their elevational distribution. J. Ecol. 110, 860–875. doi: 10.1111/1365-2745.13848

[B74] Sardans J. Penuelas J. (2014). Climate and taxonomy underlie different elemental concentrations and stoichiometries of forest species: the optimum “biogeochemical niche. Plant Ecol. 215, 441–455. doi: 10.1007/s11258-014-0314-2, PMID: 25983614 PMC4430814

[B75] Saura-Mas S. Lloret F. (2007). Leaf and shoot water content and leaf dry matter content of Mediterranean woody species with different post-fire regenerative strategies. Ann. Bot-London 99, 545. doi: 10.1093/aob/mcl284, PMID: 17237213 PMC2802959

[B76] Sides C. B. Enquist B. J. Ebersole J. J. Smith M. N. Henderson A. N. Sloat L. L. (2014). Revisiting Darwin’s hypothesis: Does greater intraspecific variability increase species’ ecological breadth? Am. J. Bot. 101, 56–62. doi: 10.3732/ajb.1300284, PMID: 24343815

[B77] Siefert A. Violle C. Chalmandrier L. Albert C. H. Taudiere A. Fajardo A. . (2015). A global meta-analysis of the relative extent of intraspecific trait variation in plant communities. Ecol. Lett. 18, 1406–1419. doi: 10.1111/ele.12508, PMID: 26415616

[B78] Umaña M. N. Zhang C. Cao M. Lin L. Swenson N. G. (2018). Quantifying the role of intra-specific trait variation for allocation and organ-level traits in tropical seedling communities. J. Veg Sci. 29, 276–284. doi: 10.1111/jvs.12613

[B79] van Bodegom P. M. Douma J. C. Verheijen L. M. (2014). Afully traits-based approach to modeling global vegetation distribution. P. Natl. Acad. Sci. U.S.A. 111, 13733–13738. doi: 10.1073/pnas.1304551110, PMID: 25225413 PMC4183343

[B80] Violle C. Enquist B. J. McGill B. J. Jiang L. Albert C. H. Hulshof C. . (2012). The return of the variance: intraspecific variability in community ecology. Trends Ecol. Evol. 27, 244–252. doi: 10.1016/j.tree.2011.11.014, PMID: 22244797

[B81] Violle C. Navas M. L. Vile D. Kazakou E. Fortunel C. Hummel I. . (2007). Let the concept of trait be functional! Oikos 116, 882–892. doi: 10.1111/j.0030-1299.2007.15559.x

[B82] Wang Y. W. He M. X. Jiang G. D. Yin P. X. Ying W. B. Yang Q. S. (2024). Characteristics of leaf functional traits of *Quercus* sp*inosa* and their responses to environmental factors at different altitude gradients in Wumeng township. Acta Exological Sin. 44, 7238–7248. doi: 10.20103/j.stxb.202308301865

[B83] Wang S. J. Li Y. B. (2007). Problems and development trends about researches on karst rocky desertification. Adv. Earth Sci. 22, 573–582. doi: 10.11867/j.issn.1001-8166.2007.06.0573

[B84] Wang J. Wang Y. He N. Ye Z. Chen C. Zang R. . (2020). Plant functional traits regulate soil bacterial diversity across temperate deserts. Sci. Total Environ. 715, 136976. doi: 10.1016/j.scitotenv.2020.136976, PMID: 32023517

[B85] Wang B. Zhang Q. Cui F. (2021). Scientific research on ecosystem services and human well-being: a bibliometric analysis. Ecol. Indic. 125, 107449. doi: 10.1016/j.ecolind.2021.107449

[B86] Wang J. Zhao W. W. Xu Z. X. Ding J. Y. Yan Y. Ferreira C. S. S. (2023). Plant functional traits explain long-term differences in ecosystem services between artificial forests and natural grasslands. J. Environ. Manage. 345, 8853. doi: 10.1016/j.jenvman.2023.118853, PMID: 37660423

[B87] Webb C. T. Hoeting J. A. Ames G. M. Pyne M. I. LeRoy Poff N. (2010). A structured and dynamic framework to advance traits-based theory and prediction in ecology. Ecol. Lett. 13, 267–283. doi: 10.1111/j.1461-0248.2010.01444.x, PMID: 20455917

[B88] Wei H. Wu B. Yang W. Luo T. (2011). Low rainfall-induced shift in leaf trait relationship within species along a semi-arid sandy land transect in northern China. Plant Biol. 13, 85–92. doi: 10.1111/j.1438-8677.2010.00321.x, PMID: 21143729

[B89] Wellstein C. Poschlod P. Gohlke A. Chelli S. Campetella G. Rosbakh S. . (2017). Effects of extreme drought on specific leaf area of grassland species: A meta-analysis of experimental studies in temperate and sub-Mediterranean systems. Global Change Biol. 23, 2473–2481. doi: 10.1111/gcb.13662, PMID: 28208238

[B90] Westerband A. Funk J. Barton K. (2021). Intraspecific trait variation in plants: a renewed focus on its role in ecological processes. Ann. Bot. 127, 397–410. doi: 10.1093/aob/mcab011, PMID: 33507251 PMC7988520

[B91] Wilson P. J. Thompson K. Hodgson J. G. (1999). Specific leaf area and leaf dry matter content as alternative predictors of plant strategies. New Phytol. 143, 155–162. doi: 10.1046/j.1469-8137.1999.00427.x

[B92] Wright S. J. (2010). The future of tropical forests. Ann. N. Y. Acad. Sci. 1195, 1–27. doi: 10.1111/j.1749-6632.2010.05455.x, PMID: 20536814

[B93] Wright I. J. Ackerly D. D. Bongers F. Harms K. E. IbarraManriquez G. Martine-Ramos M. . (2007). Relationships among ecologically important dimensions of plant trait variation in seven neotropical forests. Ann. Bot. 99, 1003–1015. doi: 10.1093/aob/mcl066, PMID: 16595553 PMC2802905

[B94] Wright I. J. Cannon K. (2001). Relationships between leaf lifespan and structural defenses in a low-nutrient, sclerophyll flora. Funct. Ecol. 15, 351–359. doi: 10.1046/j.1365-2435.2001.00522.x

[B95] Wright I. J. Dong N. Maire V. Prentice I. C. Westoby M. Díaz S. . (2017). Global climatic drivers of leaf size. Science. 357, 917–921. doi: 10.1126/science.aal4760, PMID: 28860384

[B96] Wright I. J. Reich P. B. Cornelissen J. H. C. Falster D. S. Groom P. K. Hikosaka K. . (2005). Modulation of leaf economic traits and trait relationships by climate. Global Ecol. Biogeogr 14, 411–421. doi: 10.1111/j.1466-822x.2005.00172.x

[B97] Wright I. J. Reich P. B. Westoby M. Ackerly D. D. Baruch Z. Bongers F. . (2004). The worldwide leaf economics spectrum. nature 428, 821–827. doi: 10.1038/nature02403, PMID: 15103368

[B98] Xu S. W. Su H. X. Ren S. Y. Hou J. H. Zhu Y. (2023). Functional traits and habitat heterogeneity explain tree growth in a warm temperate forest. Oecologia 203, 371–381. doi: 10.1007/s00442-023-05471-1, PMID: 37910255

[B99] Xu H. Wang H. Prentice I. C. Harrison S. P. Wang G. Sun X. (2021). Predictability of leaf traits with climate and elevation: a case study in Gongga Mountain, China. Tree Physiol. 41, 1336–1352. doi: 10.1093/treephys/tpab003, PMID: 33440428 PMC8454210

[B100] Yang B. Li J. Yan J. Zhang K. Ouyang Z. Lu Y. . (2023). Non-specific phospholipase C4 hydrolyzes phosphosphingolipids and phosphoglycerolipids and promotes rapeseed growth and yield. J. Integr. Plant Biol. 65, 2421–2436. doi: 10.1111/jipb.13560, PMID: 37642157

[B101] Yao H. F. Lu J. Wang C. Chen K. (2020). Population structure and quantitative dynamics of *pinus armandii* in bomi gang township nature reserve. For. Resour. Manage. 5, 108–115. doi: 10.13466/j.cnki.lyzygl.2020.05.016

[B102] Yao L. J. Xu Y. Wu C. P. Deng F. Y. Yao L. Ai X. R. . (2023). Variation in the functional traits of forest vegetation along compound habitat gradients in different climatic zones in China. Forests 14, 1232. doi: 10.3390/f14061232

[B103] Yuan Z. Chen H. Y. H. (2009). Global trends in senesced-leaf nitrogen and phosphorus. Glob. Ecol. Biogeogr. 18, 532–542. doi: 10.1111/j.1466-8238.2009.00474.x

[B104] Zhang H. Cao Y. G. Wang Z. J. Ye M. X. Wu R. L. (2023). Functional mapping of genes modulating plant shade avoidance using leaf traits. Plants 12, 608. doi: 10.3390/plants12030608, PMID: 36771692 PMC9920004

[B105] Zhang A. Li X. Wu S. Li L. Jiang Y. Wang R. . (2021). Spatial pattern of C:N:P stoichiometry characteristics of alpine grassland in the Altunshan Nature Reserve at North Qinghai-Tibet Plateau. Catena 207, 105691. doi: 10.1016/j.catena.2021.105691

[B106] Zhang M. Luo Y. Yan Z. Chen J. Eziz A. Li K. . (2019). Resorptions of 10 mineral elements in leaves of desert shrubs and their contrasting responses to aridity. J. Plant Ecol. 12, 358–366. doi: 10.1093/jpe/rty034

[B107] Zhang S. Zhang Y. U. Xiong K. Yu Y. Min X. (2020). Changes of leaf functional traits in karst rocky desertification ecological environment and the driving factors. Global Ecol. Conserv. 24, e01381. doi: 10.1016/j.gecco.2020.e01381

[B108] Zhao W. Reich P. B. Yu Q. Zhao N. Yin C. Zhao C. . (2018). Shrub type dominates the vertical distribution of leaf C: N: P stoichiometry across an extensive altitudinal gradient. Biogeosciences 15, 2033–2053. doi: 10.5194/bg-15-2033-2018

[B109] Zhao N. Yu G. He N. Xia F. Wang Q. Wang R. . (2016). Invariant allometric scaling of nitrogen and phosphorus in leaves, stems, and fine roots of woody plants along an altitudinal gradient. J. Plant Res. 129, 647–657. doi: 10.1007/s10265-016-0805-4, PMID: 26943163

